# Paneth cell granule dynamics on secretory responses to bacterial stimuli in enteroids

**DOI:** 10.1038/s41598-019-39610-7

**Published:** 2019-02-25

**Authors:** Yuki Yokoi, Kiminori Nakamura, Tsukasa Yoneda, Mani Kikuchi, Rina Sugimoto, Yu Shimizu, Tokiyoshi Ayabe

**Affiliations:** 1 Innate Immunity Laboratory, Graduate School of Life Science, Hokkaido University, Kita-21, Nishi-11, Kita-ku, Sapporo, Hokkaido 001-0021 Japan; 2 Department of Cell Biological Science, Faculty of Advanced Life Science, Hokkaido University, Kita-21, Nishi-11, Kita-ku, Sapporo, Hokkaido 001-0021 Japan

## Abstract

Paneth cells at the base of small intestinal crypts secrete granules containing α-defensins in response to bacteria and maintain the intestinal environment by clearing enteric pathogens and regulating the composition of the intestinal microbiota. However, Paneth cell secretory responses remain debatable and the mechanisms that regulate the secretion are not well understood. Although enteroids, three-dimensional cultures of small intestinal epithelial cells, have proven useful for analyzing intestinal epithelial cell functions including ion transport, their closed structures have imposed limitations to investigating interactions between Paneth cells and the intestinal microbiota. Here, we report that microinjection of bacteria or lipopolysaccharide (LPS) into the enteroid lumen provides an *ex vivo* system for studying Paneth cell secretion in real-time. The results show that Paneth cells released granules immediately when the apical surfaces of enteroid epithelial cells were exposed to LPS or live bacteria by microinjection. However, Paneth cells did not respond to LPS delivered in culture media to enteroid exterior basolateral surface, although they responded to basolateral carbamyl choline. In addition, Paneth cells replenished their granules after secretion, enabling responses to second stimulation. These findings provide new insight for apically-induced Paneth cell secretory responses in regulating the intestinal environment.

## Introduction

The small intestine absorbs luminal nutrients and also provides innate mucosal immune mechanisms that protect and prevent infection and invasion by certain pathogens^1–4^. Epithelial cells that line the small intestine form a barrier consisting of intestinal epithelial stem cells (ISCs) and four major lineages of differentiated cells, including absorptive enterocytes, enteroendocrine cells, goblet cells, and Paneth cells that are oriented along the villus-crypt axis^5^. Paneth cells, which occupy the base of small intestinal crypts with Lgr5^+^ ISCs, contribute to innate enteric immunity by releasing secretory granules rich in varied host defense peptides, e.g., α-defensins, in response to bacteria and bacterial antigens such as lipopolysaccharide (LPS)^6–9^. On the contrary, it was reported that Paneth cells do not respond to luminal bacterial antigens directly but that an uncharacterized immune cell releases interferon gamma (IFN-γ) and that IFN-γ is what stimulates Paneth cell secretion^10^. Therefore, Paneth cell secretory responses to bacterial stimuli have been controversial.

More than 1 × 10^14^ bacteria live in the human intestinal lumen and harmonize with the host to create a normal intestinal microbiota of symbiotic microorganisms that contribute to maintaining intestinal homeostasis^11–14^. Disruption of the intestinal microbiota induces dysbiosis and is associated with various diseases such as inflammatory bowel disease, obesity and diabetes mellitus^15–19^. In *DEFA5*^+/+^ mice, which express the human enteric α-defensin HD5 transgene in Paneth cells, the ileal microbiota contained significantly fewer Firmicutes and a higher percentage of Bacteroidetes compared to their wild-type FVB littermates^20^. In contrast, the ileal microbiota of matrix metalloproteinase 7-null (*Mmp7*^−/−^) mice, which are deficient in activated luminal α-defensins (termed cryptdins), had a significantly greater percentage of Firmicutes and fewer Bacteroidetes compared to wild-type control mice^20^. α-Defensins secreted into the small intestinal lumen have been recovered as intact, functional forms in the lumen of large intestine^21,22^. Furthermore, we showed that active cryptdins are detected not only in luminal contents of the intestine, but also in feces^23^, suggesting that α-defensins also may influence the composition of the large intestinal microbiota. Masuda *et al*. showed that cryptdin-4 had potent *in vitro* bactericidal activities against pathogenic bacteria and less activity against commensal species, suggesting that the peptide may regulate the composition of the intestinal microbiota^24^. Taken together, secreted Paneth cell α-defensins have a role in regulating the intestinal microbiota and thus contribute to intestinal homeostasis. Moreover, Paneth cell dysfunction is associated with certain diseases such as inflammatory bowel disease, obesity and enteropathy in graft-versus-host disease (GVHD)^25^. In GVHD model mice, loss of secreted α-defensins due to depletion of Paneth cell numbers is associated with subsequent dysbiosis, resulting in fatal sepsis^26,27^. Furthermore, administered α-defensin partially prevents dysbiosis and improves GVHD survival^28^. These reports suggest that dysfunction of Paneth cell α-defensin secretion is a major factor in initiating dysbiosis and disease and that secretion of Paneth cell granules is a key contributor to maintaining the *in vivo* intestinal environment via controlling the intestinal microbiota. However, mechanisms that regulate Paneth cell granule secretion remain undefined, partly because quantitative *ex vivo* methods of evaluating secretion have not been applied to the problem.

The culture of intestinal epithelial cells and their growth and differentiation into three dimensional enteroids provides an intact system consisting of stem cells and all intestinal epithelial cell lineages, including Paneth cells, oriented along crypt projections that protrude from a large central lumen^29^. Enteroids have been adapted to study physiological functions such as nutrient absorption, hormone secretion, ion and drug transport of intestinal epithelial cells^30–32^. Although enteroids may be adapted for analysis of Paneth cell function, their exposure to secretory stimuli in culture media is limited to the basolateral epithelial surfaces, because enteroids are closed structures. To resolve this limitation and to deliver agonists to the enteroid lumen, we introduced test substances to the lumen of enteroids by microinjection. In this study, the *ex vivo* enteroids enabled us to visualize and quantify Paneth cell granule secretion in response to LPS and live bacteria and to show that Paneth cells responded only to apical bacterial stimuli. Also, we observed the restoration of Paneth cell homeostasis by showing that Paneth cells replenish their granule content *de novo* within 21 hours after secretion and release the resynthesized granules upon secondary stimulation.

## Results

### Basolateral exposure of enteroids to LPS does not stimulate Paneth cell secretion

**Figure 1:**
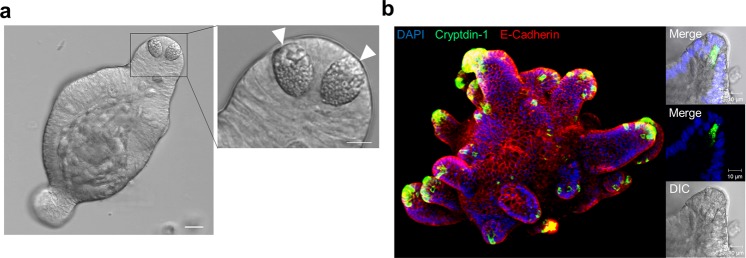
Expression of α-defensin in Paneth cells of enteroids. (**a**) DIC images of Paneth cells at the bottom of crypt-like structure in enteroid at day 3 (white arrows) acquired by using confocal laser microscopy. Scale bar: 10 μm. (**b**) Immunofluorescent staining of mouse α-defensin, cryptdin-1 (green) contained in the granules of Paneth cells. E-cadherin (red) as an epithelial cell marker and nuclei (blue). Left: Z-stack image of enteroid. Right: High-magnification images of Paneth cells at the bottom of crypt-like structure.

Paneth cells in enteroids cultured from isolated small intestinal crypts were observed by confocal laser scanning microscopy with differential interference contrast (DIC) imaging. Paneth cells were localized at the base of crypt-like structures that protrude from the enteroid lumen and contain granules with mean diameters of approximately 1 μm (Fig. 1a). To test whether enteroid Paneth cells express α-defensins at levels and with the same specificity known for Paneth cells *in vivo*, immunostaining experiments were conducted using a monoclonal antibody to cryptdin-1 (Crp1, Defa1). Crp1 immune reactivity was restricted to Paneth cells at the bottoms of crypt-like structures (Fig. 1b, left), and high-resolution images showed that Crp1 was localized in Paneth cell secretory granules (Fig. 1b, right). These results confirmed that the α-defensin Crp1 is an abundant constituent of enteroid Paneth cells, where it accumulates in secretory granules.

**Figure 2:**
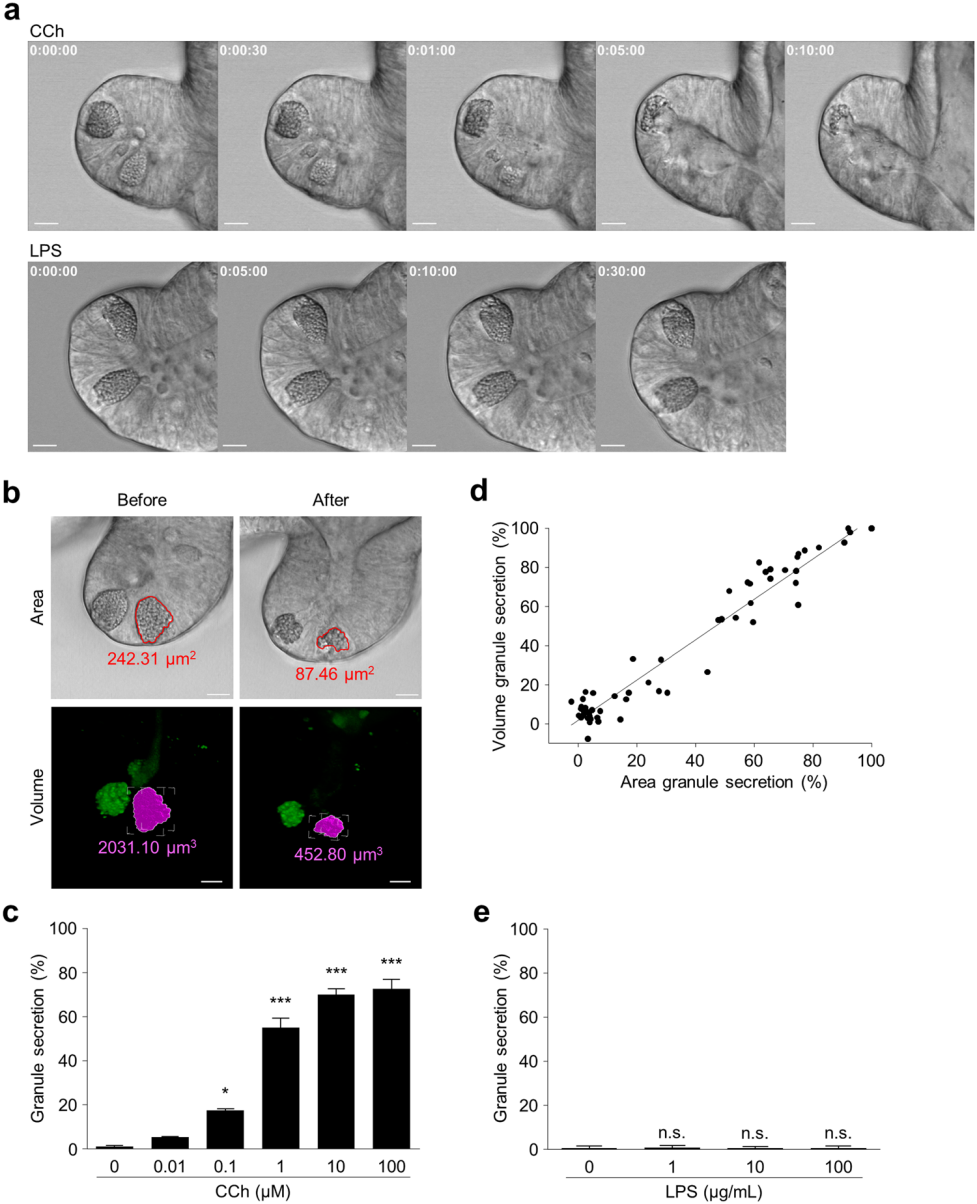
Paneth cell granule secretion in response to basolateral stimuli. (**a**) Representative time-lapse images of Paneth cells obtained by confocal laser microscopy after adding 10 μM CCh or 100 µg/mL LPS from *S*. Typhimurium to the culture medium of three independent experiments for each. Real acquisition time are represented by h:mm:ss (hour:minute:second). Scale bars: 10 μm. (**b**) Representative area of granules (red line, top) and volume of granules (pink, bottom) in Paneth cells before and 30 min after 10 μM CCh stimulation. Scale bar: 10 μm. (**c**) Percent granule secretion calculated as percent area granule secretion when Paneth cells in enteroid were stimulated with 0–100 μM CCh for 30 min. 0 μM: 1.07 ± 0.33%; 0.01 μM: 5.26 ± 0.20%; 0.1 μM: 17.52 ± 0.51%; 1 μM: 54.79 ± 4.45%; 10 μM: 69.88 ± 2.56%; 100 μM: 72.49 ± 4.31%. (**d**) Correlation analysis between percent area granule secretion and percent volume granule secretion by 0–100 μM CCh (*P* < 0.0001, r = 0.90). The data were obtained from ten Paneth cells in five enteroids for each concentration. (**e**) Percent granule secretion of Paneth cells 30 min after adding 1–100 μg / mL LPS from *S*. Typhimurium to the medium. 0 µg/mL: 0.46% ± 0.25%; 1 µg/mL: 0.72 ± 0.27%; 10 µg/mL: 0.38 ± 0.26%; 100 µg/mL: 0.54% ± 0.07%. The values of (**d**,**e**) were depicted as mean ± standard error of the mean for three independent experiments. *P < 0.05, ***P < 0.0001 vs. control; n.s., not significant.

Carbamyl choline (carbachol, CCh) is a cholinergic agonist that stimulates α-defensin secretion from Paneth cells^7,33^, and enteroids exposed to carbachol were analyzed by time-lapse DIC imaging using confocal microscopy to visualize the secretory capability of these *ex vivo* Paneth cells. Immediately upon addition of 10 μM CCh to culture medium, granule secretion toward the enteroid lumen was initiated. Apical granules were released first, and the majority of granules was secreted into the lumen within 10 min of CCh exposure (Fig. 2a, Supplementary Video S1). In sharp contrast, no granule secretion was detected by Paneth cells exposed basolaterally to LPS by addition to the culture medium (Fig. 2a).

Confocal microscopic analysis of DIC images allowed the area within Paneth cells occupied by granules to be measured, and that area decreased after CCh-induced secretion (Fig. 2a, Supplementary Video S1). Granule area in Paneth cells was measured using DIC images before and 30 min after addition of up to 100 μM CCh (Fig. 2b, top), and the percent of secretory granule area released was calculated. Percent granule area secreted by enteroid Paneth cells in response to CCh increased in concentration-dependent manner (Fig. 2c).

Because Paneth cells are 3D structures, we next determined whether the percent of granule area secreted as calculated by analysis of 2D images was an accurate reflection of Paneth cell granule secretion. Paneth cell granules were labeled with Zinpyr-1, a fluorescent, membrane-permeable, Zn^2+^ chelator^34^, owing to substantial stores of Zn^2+^ in Paneth cell granules, and the volume of Paneth cell granules before and after CCh stimulation was measured by analysis of 3D images (Fig. 2b, bottom). There was a strong positive correlation between the percent area of granule secretion and the percent volume of granule secretion (Fig. 2d, r = 0.90, p < 0.0001). Accordingly, dose-dependent CCh-induced Paneth cell secretion was assessed by percent area, a simpler index of equal accuracy (Fig. 2c). In contrast to the robust secretory response to basolateral CCh, no granule release occurred when LPS was added to the culture medium (Fig. 2e), consistent with evidence that Paneth cells do not recognize LPS basolaterally^10^.

### Introduction of stimuli into the enteroid lumen by microinjection

**Figure 3:**
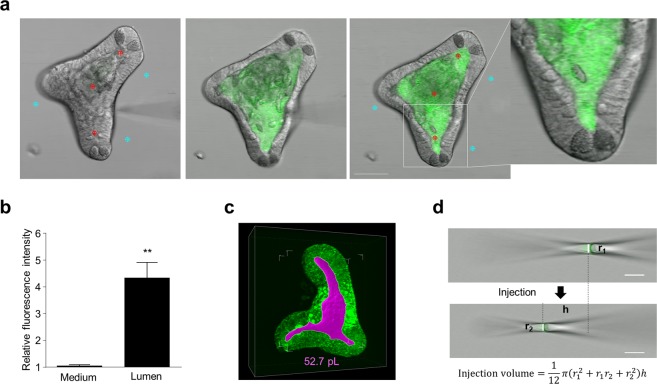
Introducing reagents into enteroid lumen by microinjection. (**a**) Time-lapse images of fluorescein microinjection into enteroid lumen. Scale bar: 50 μm. (**b**) Relative Fluorescence intensities of enteroid lumen (Red ROI, 4.30 ± 0.57) and of medium (Blue ROI, 1.05 ± 0.01) at post-injection (after 5 min) against pre-injection calculated from six enteroids injected fluorescein. The values are depicted as mean ± standard error of the mean. **P < 0.001. (**c**) Representative Z-stack image of enteroid obtained at 180 min after adding 1 μM Rh123 into the culture medium (Pink: lumen). (**d**) The formula for calculating injection volume from inner diameter (r_1_, r_2_) and moving distance (h) of liquid interface of mineral oil and fluorescein in the needle at before (upper) and after (under) microinjection. Scale bar: 10 µm.

Because the intestinal microbiota and their antigens are present in the intestinal lumen, *in vivo*, Paneth cells are exposed to these stimuli from the apical side. Accordingly, to ascertain whether apical exposure of enteroid Paneth cells to LPS would induce secretion, we used microinjection to deliver substances to the enteroid lumen. Enteroids with 2–3 crypt protrusions had adequate luminal volume for needle insertion while still providing for effective imaging of Paneth cell responses to microinjected agents (Fig. 3a, left). Fluorescent substances microinjected into the enteroid lumen were evident at the Paneth cell apical surfaces and were retained. Fluorescein was introduced as a visual tracer via needle inserted at a position between crypt-like structure and villus-like lumen (Fig. 3a, left). Fluorescence intensity increased only within the enteroid lumen, and reached the apical surfaces of enteroid Paneth cells immediately (Fig. 3a, right, Supplementary Video S2). Enteroid integrity was maintained under these conditions, as shown by measurements of luminal and basolateral fluorescence intensities before and after microinjection. For example, after microinjection, luminal fluorescence intensities were elevated significantly (4.30 ± 0.57), but no change in basolateral fluorescence intensity (1.05 ± 0.01) was observed in the enteroid exterior (Fig. 3a,b). These results showed that microinjected agents were retained and that enteroid integrity was not compromised by the microinjection procedure.

To determine the effective concentration of substances microinjected into the lumen, the volume of the enteroid lumen and sample volume injected via the microneedle were measured. Rhodamine123 (Rh123) is a fluorescent dye and a known P-glycoprotein substrate that is transported directionally from the basolateral to apical surface and accumulates in the luminal space^32^. Accordingly, we stained the lumen of enteroids with Rh123 by addition to culture media, and luminal volumes were measured at 54.4 ± 3.0 pL (Supplementary Table S1) from Z-stack images (Fig. 3c, Supplementary Video S3). In addition, we calculated the volume of microinjected agents to be 2.5 ± 0.1 pL, based on the diameter (r) of the interface between the fluorescein and mineral oil in the microneedle at before (r_1_) and after (r_2_) microinjection and the distance (h) the interface traveled during microinjection (Fig. 3d, Supplementary Table S1). Thus, the concentration of substances that were microinjected underwent a 21.5 ± 1.2-fold dilution by injection into the enteroid lumen (Supplementary Table S1). These considerations established a novel system for evaluating the effects of introducing agents into the enteroid lumen by microinjection on Paneth cell secretory responses.

### Apical exposure of Paneth cells to LPS or live bacteria induces secretion

**Figure 4:**
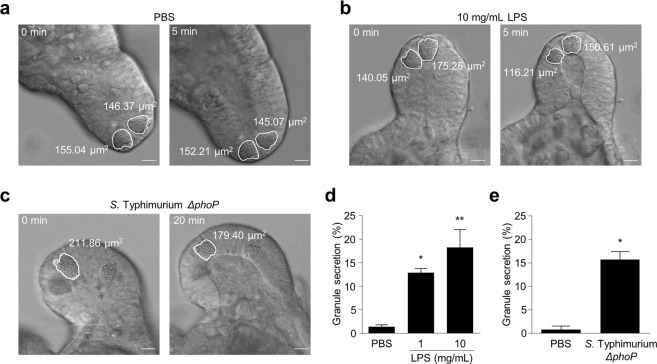
Paneth cell granule secretion in response to apical LPS and *S*. Typhimurium stimuli. Representative time-lapse images before and after microinjection of (**a**) PBS, (**b**) 10 mg/mL LPS from *S*. Typhimurium and (**c**) 1.0 × 10^10^ CFU/mL *S*. Typhimurium *ΔphoP*. Scale bars: 10 μm. (**d**) Percent granule secretion of Paneth cells 5 min after introducing 1–10 mg/mL LPS from *S*. Typhimurium into enteroid lumen. PBS: 1.32 ± 0.46%, 1 mg/mL: 12.85 ± 0.90%, 10 mg/mL: 18.27 ± 2.19%. (**e**) Percent granule secretion of Paneth cells 20 min after introducing *S*. Typhimurium *ΔphoP* into enteroid lumen. PBS: 0.76 ± 0.75%, 1 × 10^10^ CFU/mL: 15.67 ± 1.74%. The values of (**d**,**e**) were depicted as mean ± standard error of the mean for three independent experiments. *P < 0.01, **P < 0.001 vs. PBS.

Paneth cells secrete granules in response to infectious stimuli delivered to the enteroid lumen by microinjection (Fig. 4a–c). After introduction of LPS to the apical surface of Paneth cells by microinjection of 1 mg/mL LPS (46.5 μg/mL luminal concentration) or 10 mg/mL LPS (465 μg/mL in the lumen), Paneth cells began secreting granules within 2–14 sec (Fig. 4b, Supplementary Video S4). At the lower LPS level Paneth cells released 12.85 ± 0.90% of their granule volume, which increased to 18.27 ± 2.19% at the higher LPS concentration, both significantly elevated compared to the Ca^2+^- and Mg^2+^-free phosphate-buffered saline (PBS) vehicle (Fig. 4d). In contrast, administration of PBS into the enteroid lumen had no stimulatory effect on Paneth cell secretion (Fig. 4a, Supplementary Video S5). Therefore, PBS vehicle or the microinjection procedure did not induce Paneth cells to secrete. In addition, when live *Salmonella enterica* serovar Typhimurium *ΔphoP* (*S*. Typhimurium *ΔphoP*) were introduced into the enteroid lumen, Paneth cells began releasing granules within 4–19 min after microinjection (Fig. 4c, Supplementary Video S6). Paneth cells responded to luminal injection of 1 × 10^10^ CFU/mL of *S*. Typhimurium *ΔphoP*, or ~4.7 × 10^8^ CFU/mL, by secretion of 15.67 ± 1.74% of their granules, which was elevated significantly compared to PBS vehicle. Thus, Paneth cells secrete granules in response to apical exposure to LPS or live bacteria but are refractory to basolateral exposure to the same agonists.

### Granule replenishment after Paneth cell secretory responses

**Figure 5:**
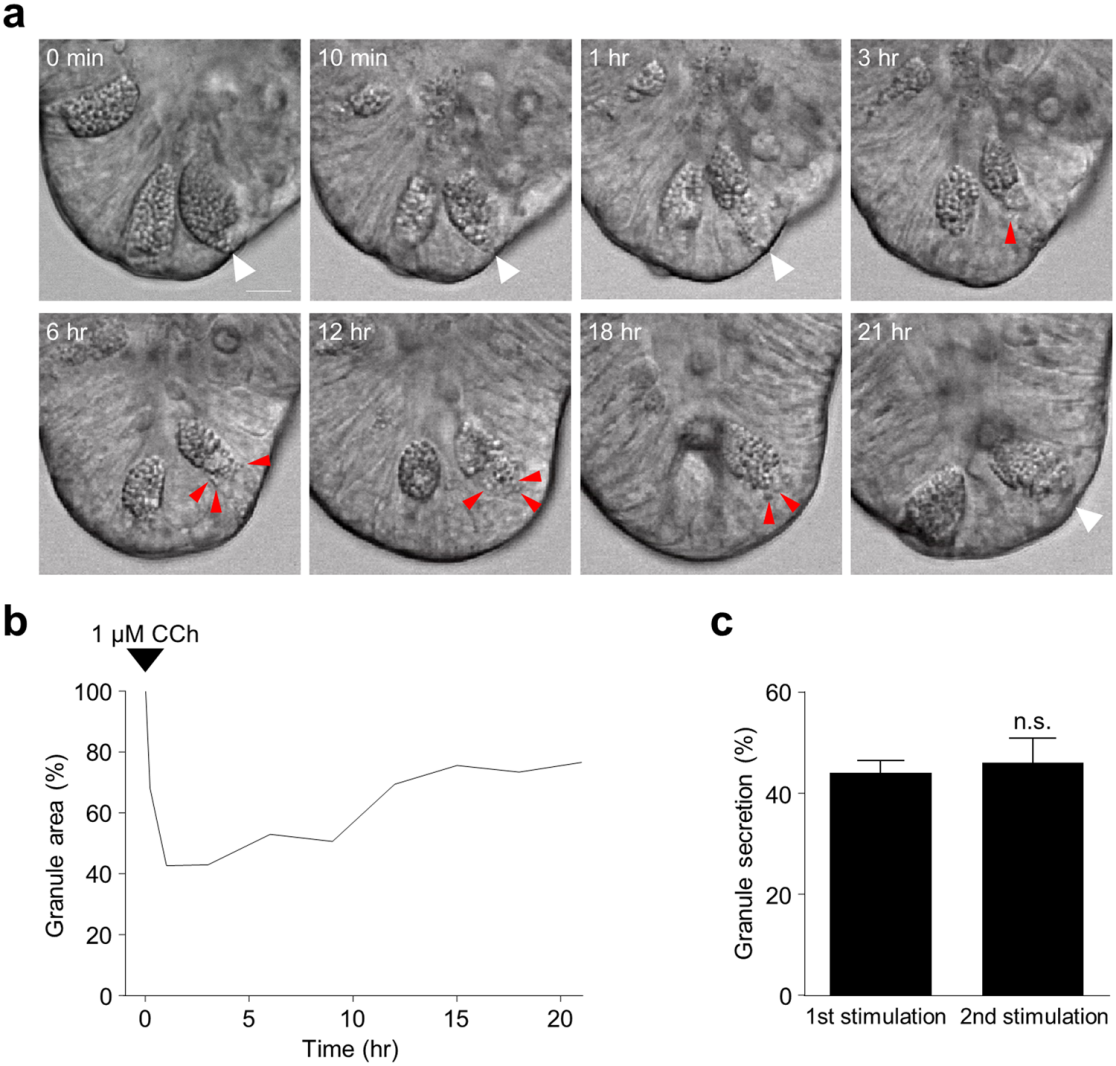
Paneth cell granulogenesis after CCh-induced granule secretion. (**a**) Time-lapse images of Paneth cell granulogenesis obtained by confocal laser microscopy. Time denotes the elapsed time from adding CCh to the medium. Paneth cells secreted their granules by 1 μM CCh for 10 minutes, then replaced with flesh medium and follow the same Paneth cell (white arrow). The newly generated granules were presented in red arrows. Scale bars: 10 μm. (**b**) The time course of Paneth cell granule area relative to before 1 µM CCh stimulation. 1 µM CCh was added to the medium at time 0 followed by CCh washed out after 10 min. 0 h: 100.0%, 0.2 h: 68.2%, 1 h: 42.7%, 3 h: 42.97%, 6 h: 53.0%, 9 h: 50.7%, 12 h: 69.5%, 15 h: 75.7%, 18 h: 73.5%, 21 h: 76.7%. (**c**) Percent granule secretion of nine Paneth cells in five enteroids stimulated with 1 µM CCh for 10 min. 1st stimulation: 43.96 ± 7.26, 2nd stimulation (after granule replenishment): 45.89 ± 14.59. The values are depicted as mean ± standard error of the mean; n.s., not significant.

To determine whether Paneth cells restore their granule contents after induced secretion, granule formation was visualized in Paneth cells by confocal microscopy analysis following secretion in response to CCh. After addition of 1 μM CCh, Paneth cell granule secretion began immediately and continued for 10 min after CCh had been washed out by change of media. Within 1 h, small dense vesicles could be seen being transported from near the nucleus toward the apical cell surface, gradually the Paneth cell volume occupied by granules was restored nearly to the original volume as new granules were synthesized *de novo* and trafficked apically (Fig. 5a, Supplementary Video S7). Replenishment of Paneth cell secretory granules continued throughout the 21 h period of observation. The granule area began to increase at 1 h and reached 76.7% of the original granule volume at 21 h after secretion (Fig. 5b). When Paneth cells with newly restored granule volume were stimulated by a second exposure to CCh as before, they responded by releasing regenerated granules in as robust a manner as upon the first CCh exposure (Fig. 5c).

## Discussion

In this study, we visualized Paneth cell secretory responses to apical bacteria and LPS which were introduced into the enteroid lumen by microinjection and quantified the extent of Paneth cell granule secretion in response to these stimuli. Further, the polarity of Paneth cell recognition of LPS and *S*. Typhimurium *ΔphoP* was revealed, because apical, but not basolateral, exposure induced granule secretion. Previously, we quantified Paneth cell secretion by isolated intact crypts exposed to bacteria and bacterial antigens by measuring secretion of α-defensins, their bactericidal activity *in vitro*, and their release using western blot analysis^7^. However, because isolated crypts are not closed structures Paneth cell exposure may have been luminal and/or basolateral, and we could not distinguish whether recognition was apical or basolateral.

In this study, Paneth cells exhibited no secretory responses to basolateral LPS exposure, but microinjection of LPS into the enteroid lumen elicited a robust, immediate, yet limited degranulation response, evidence of apical Paneth cell recognition of LPS. Farin *et al*. evaluated Paneth cell responses to bacterial stimuli by detecting enteroid lysozyme levels using intact enteroids, which only allowed basolateral exposure to be compared with combined basolateral and apical exposure of mechanically sheared enteroid fragments. Because Paneth cells did not respond to either basolateral and apical LPS stimulation, Paneth cell secretory responses in freshly isolated intestinal crypts were hypothesized to depend on co-isolated intraepithelial leukocytes^10^. Here, by utilizing a high-sensitivity system to visualize granule release and quantify secretion, Paneth cells were shown to respond almost immediately after LPS microinjection. In contrast, we have shown that T cell-derived cytokine, IFN-γ induces Paneth cell death through a caspase-3/7-dependent pathway within 13 to 16 h of IFN-γ exposure to enteroids^35^, consistent with previous reports^10^. Our results indicate that the rapid response of Paneth cells to luminal LPS exposure is clearly distinct from the effects of IFN-γ and that Paneth cells sense LPS directly at their apical surface. Because Paneth cells do not express Toll-like receptor 4 (TLR4), the receptor for LPS, Paneth cells recognize LPS and secrete granules via a TLR4-independent pathway^9,36–39^. Consistent with this evidence, Paneth cells in crypts from C3H/HeJ mice, which lack functional TLR4, show the same secretory responses as WT mice^9^. Furthermore, although quantifying precise final concentrations of substances introduced into the enteroid lumen is difficult because luminal volumes of individual enteroids vary, we found that reliable concentrations could be calculated for enteroids cultured for 3 days. As a result, we confirmed that estimated final concentration of LPS inducing Paneth cell granule secretion was consistent with previous reports^7,9^. We further clarified that Paneth cells sense Gram-negative *S*. Typhimurium *ΔphoP* apically and release granules. Although the overall enteroid bactericidal activities after luminal introduction of *S*. Typhimurium into enteroids have been determined^40^, Paneth cell secretory responses were not characterized. By visualizing Paneth cell granule secretion with high resolution, we confirmed that Paneth cells secrete α-defensins in response to live apical *S*. Typhimurium *ΔphoP* and that the initiation of secretion in response to live bacteria was slower than responses to LPS. Also, orally administered *S*. Typhimurium survive in greater numbers in *Mmp7*^−/−^ mice compared to wild-type mice^41^. Thus, our results are consistent with the finding that Paneth cells contribute to innate enteric immunity by releasing α-defensins in response to orally ingested pathogenic bacteria that reach the small intestine directly. Also, the microinjection system enabled disclosure of previously unknown Paneth cell dynamics and mechanisms of Paneth cell granule secretion and resynthesis. Varied bacterial antigens including Toll-like receptor ligands such as lipoteichoic acid and CpG oligodeoxynucleotides and NOD2 ligands such as muramyl dipeptide induce Paneth cell secretion^7,9,42^. Our results indicated that Paneth cells secrete granules containing α-defensins by recognizing luminal bacterial antigens. Further studies are needed to clarify if Paneth cells secrete granules in response to other Gram-negative bacteria or Gram-positive bacteria which reside in the intestine in addition to *S*. Typhimurium.

Muscarinic nerve stimuli and cytokines such as IL-13 and IL-22 which are delivered at the basolateral surface of Paneth cells also induce granule secretion^43–45^. In this study, we have shown dose-dependent Paneth cell secretion in response to CCh by basolateral exposure. These results are consistent with previous findings of CCh-induced α-defensin secretion using isolated crypts of mouse small intestine^7^ and confirm that Paneth cells elicit a dynamic secretory response by sensing cholinergic stimulation basolaterally. Our findings further suggest that Paneth cells may secrete granules by recognizing agonists in the basolateral environment, including cytokines and neuro-transmit factors via crosstalk among immune cells and nerve cells^43–45^. Taken together, Paneth cells appear to mediate innate immunity via granule secretion in response to complex stimuli on both the luminal and basolateral surfaces of the small intestinal epithelium.

The intestinal lumen is chronically exposed to microorganisms, including pathogens, that are introduced orally. Therefore, the fate of Paneth cells after secreting granules, i.e., whether they remain depleted, undergo apoptosis, or replenish granules in preparation for additional microbial exposure is a key element in their life cycle. Although it was reported that secreted synaptic vehicles from the synaptic terminal of neurons are recycled^46,47^, the regeneration of secretory granules after release from Paneth cells has not been explored previously. We have found that Paneth cells, after depleting their granule stores by secretion in response to CCh, become refilled with newly generated granules *ex vivo* within 21 h. In addition, Paneth cells that have resynthesized their granule contents respond to a secondary CCh stimulus with a comparably robust release of granules, evidence that individual Paneth cells prepare for multiple microbial exposures in contributing to innate enteric immunity. The dynamics of granulogenesis observed at high resolution visualized vesicular transport starting from the periphery of the Golgi near the nucleus toward the apical Paneth cell surface, within 21 h of primary CCh stimulation. These findings suggest that Paneth cells also may repeatedly secrete and replenish their secretory granules *in vivo* to contribute continuously to innate host defense against luminal enteric microorganisms.

In conclusion, by establishing a novel *ex vivo* evaluation system to visualize and quantify Paneth cell granule secretion, we determined that interactions between Paneth cells and bacteria and their antigens occur only on the apical Paneth cell surface, disclosing previously unknown interactions in the intestinal environment. In certain diseases, Paneth cells have been identified as the site of susceptibility gene defects that disrupt the secretion of Paneth cell granules. For example, mutations in the Crohn’s Disease susceptibility genes, *Atg16l1* and *Xbp1* result in abnormal granule morphology and reduced numbers of granules in Paneth cells both in genetically deficient mice and in Crohn’s disease patients^48,49^. Furthermore, expression levels of HD5 and lysozyme in Paneth cell granules of obese patients are decreased significantly compared to healthy subjects of normal weight^50^. Because α-defensins regulate the composition of the intestinal microbiota^28,51^, refining the understanding of Paneth cell granule secretion is vital to reveal pathology of such diseases. The physiological dynamics that occur in Paneth cells as revealed in this study may lead to an understanding of mechanisms that maintain intestinal homeostasis and further contribute to development of preventive medicine or therapeutics for diseases involving disruption of Paneth cell biology and their α-defensins.

## Methods

### Mice

C57BL/6 mice and Crlj: CD1 (ICR) mice were purchased from CLEA Japan and from Charles River Japan, respectively. Enteroids for Paneth cell granule secretion analyses were derived from C57BL/6 mice. ICR mice were used to generate enteroids for immunofluorescence staining. All animal experiments were approved by the committee on animal experimentation at Hokkaido University. All experiments were performed in accordance with relevant guidelines and regulations of Hokkaido University.

### Small intestinal crypt isolation and enteroid culture

For crypts isolation, proximal 5 cm of small intestine was removed from adult mice euthanized by cervical dislocation. Mouse small intestine was flushed with ice-cold PBS and cut open lengthwise. The villi were scraped off using a scalpel blade, and remaining tissue was incubated in 30 mM EDTA with Ca^2+^- and Mg^2+^-free Hank’s balanced salt solution (HBSS) for 10 min at room temperature. The tissue was shaken vigorously ~100 times in fresh HBSS to exfoliate crypts followed by transfer to fresh HBSS and the shaking step repeated 4 times. Each fraction was centrifuged at 440 *g* for 4 min at 4 °C and isolated crypts were resuspended in HBSS supplemented with 10 µM Y-27632 (Sigma-Aldrich). After counting the crypts of each fraction, the fraction with >80% purity of crypts was centrifugated at 400 *g* for 4 min at 4 °C. A total of 150 crypts were mixed with 30 μL of Matrigel (Corning) and plated in 48-well plates (Thermo Fisher Scientific). After polymerization of Matrigel, 250 μL of enteroid culture medium composed of Advanced DMEM/F12 containing penicillin/streptomycin, 10 mM HEPES, 1× GlutaMAX, 1× N2, 1× B27 (all from Thermo Fisher Scientific), 1 μM N-acetylcysteine (Sigma-Aldrich), 50 ng/mL EGF (Peprotech), 50 ng/mL HA-R-Spondin1-Fc (produced using Cultrex^®^ HA-R-Spondin1-Fc 293T Cells, #3710-001-01, Trevigen) and 100 ng/ml Noggin (Peprotech) was overlaid. Culture medium was changed every other day.

### Immunofluorescence staining

For enteroid immunofluorescence staining, Matrigel was mechanically disrupted by pipetting, and enteroids were transferred onto Lab-TeK^TM^ II Chamber Slide (Thermo Fisher Scientific). Enteroids were fixed with 4% paraformaldehyde, followed by permeabilization and blocking with 0.1% Triton X-100 and 5% goat serum in PBS for 30 min at room temperature. Primary antibody reaction was performed with 10 µg/mL Rat monoclonal anti-cryprdin-1 (clone: 77-R63, produced by our laboratory) and 5 µg/mL mouse monoclonal anti-E-cadherin (clone: 36/E-Cadherin, BD Transduction Laboratories) diluted by 1% Triton X-100 and 10% Block Ace (DS Pharma Biomedical) in PBS (antibody dilution buffer) for 2 h at room temperature. Enteroids incubated with Alexa Fluor 488 goat anti-Rat IgG and Alexa Fluor 594 goat anti-Mouse IgG (dilution 1:400, Thermo Fisher Scientific) diluted by antibody dilution buffer for 1 h at room temperature. Enteroids were also counterstained with 5 µg/mL staining 4′6-Diamidino-2-phenylindole (DAPI, Thermo Fisher Scientific) for 5 min at room temperature to visualize nuclei and were mounted with Aqua Poly/Mount (Polysciences). Pictures were taken using a confocal microscope (LSM 510 META, Carl Zeiss).

### Reagents to stimulate Paneth cells

CCh (Sigma-Aldrich) was dissolved in Advanced DMEM/F12 to prepare 10 mM solution at time of use and the stock solution was diluted with enteroid culture medium to final concentration. Fluorescein (1% NH2-Reactive Fluorescein) was prepared from NH2-Reactive Fluorescein in Fluorescein Labeling Kit - NH2 (Dojindo Laboratories) which was dissolved in 10 μL dimethyl sulfoxide followed by 100X dilution with PBS. LPS from *S*. Typhimurium purified by phenol-chloroform extraction following gel-filtration chromatography (L2262, Sigma-Aldrich) was dissolved in PBS to prepare 10 mg/mL stock solution and was sonicated for 30 min before use.

### Visualization and quantification of Paneth cell granule secretion and replenishment

Enteroids at day 3 of culture were harvested by dissolving Matrigel including organoids with cold PBS and were transferred onto collagen-coated 8 well chamber cover (Matsunami) at 100 enteroids/well. The chamber was stood on ice for 5 min to sink enteroids to the bottom. Matrigel was polymerized followed by adding enteroid culture media pre-warmed to 37 °C. The chamber was fixed on Stage Top Incubator (Tokai Hit) which was thermally-controlled enclosure set at 37 °C, 5% CO_2_. The DIC images of Paneth cells before and after stimulation were acquired by using a confocal microscope (A1, Nikon) equipped with 0.95 NA objective lens (CFI Apo LWD 20X WI λS, Nikon) and resonant scanner at 15 frame/sec. Tracing outer edge of Paneth cell granule discriminated by contrast of the DIC images using 13HD Creative Pen Display (Wacom), Paneth cell granules area was surrounded at pre- and post-stimulation and area of the granules was measured by using an image analysis software, NIS-Elements AR (Nikon). Percent area granule secretion was expressed as below: Equation (1)

1 $${\rm{Granule}}\,{\rm{secretion}}\,( \% ,{\rm{area}})=({\rm{1}}-\frac{{\rm{Granule}}\,{\rm{area}}\,\mathrm{post} \mbox{-} \mathrm{stimulation}}{{\rm{Granule}}\,{\rm{area}}\,\mathrm{pre} \mbox{-} \mathrm{stimulation}})\times {\rm{100}}$$

Ten Paneth cells in five enteroids were analyzed for each concentration. For quantification of Paneth cell granule volume, Paneth cell granules were labeled by addition 10 µM Zinpyr-1 (Santa Cruz Biotechnology) to the culture medium for 16 h at 37 °C, 5% CO_2_ and transferred to 8 well chamber cover. Z-stack images of top to lower end of Paneth cell granules were acquired with 1 µm Z-step before and after 30 min CCh stimulation. The green fluorescence signal at 488 nm was detected by a GaAsP PMT. Volume of Zinpyr-1^+^ region in Paneth cell was measured by using 3D measurement tool in NIS-Elements AR. Percent volume granule secretion was expressed as below: Equation (2)

2 $${\rm{Granule}}\,{\rm{secretion}}\,( \% ,\mathrm{volume})=({\rm{1}}-\frac{{\rm{Granule}}\,{\rm{volume}}\,\mathrm{post} \mbox{-} \mathrm{stimulation}}{{\rm{Granule}}\,{\rm{volume}}\,\mathrm{pre} \mbox{-} \mathrm{stimulation}})\times {\rm{100}}$$

To determine Paneth cell granule refilling, enteroids were stimulated with 1 µM CCh for 10 min and enteroid culture medium containing CCh was washed out by prewarmed Advanced DMEM/F12 at three times and replaced fresh enteroid culture medium. To capture the images in focus on granules, Z-stack time-lapse images of Paneth cell granule refilling were acquired with 1 µm Z-step at 15 min intervals for 21 h at low laser power. After granule refilling, Paneth cells were stimulated with 1 µM CCh again for 10 min, and percent granule secretion was calculated using replenished granule area.

### Bacterial culture

The *phoP-phoQ* regulon of Salmonellae regulates numerous genes that results in sensitivity to endogenous cationic antimicrobial peptides, including α-defensins, and *phoP* null strain of *S*. Typhimurium is sensitive to α-defensins^7,9^. We used a defensin-sensitive *ΔphoP* strain of *S*. Typhimurium to enhance the sensitivity of our determinations. *S*. Typhimurium *ΔphoP* was pre-cultured in 3 mL of tryptic soy broth (Becton Dickinson) for 16 h followed by growing to an OD_600_ of 0.6–0.8 in 30 mL of tryptic soy broth at 37 °C with shaking at 180 rpm. Exponential-phase bacteria were deposited by centrifugation at 3,000 *g* at 4 °C for 10 min. Bacteria were washed once and resuspended in ice-cold PBS to a concentration of 1 × 10^10^ CFU/mL.

### Enteroid lumen microinjection

Enteroids at day 3–4 of culture were transferred onto Cell Imaging Dish (eppendorf) at 100 enteroids/dish as same as the above-mentioned method. For *S*. Typhimurium *ΔphoP* injection, the culture medium without antibiotics was used. Microinjection was performed while scanning enteroid at 15 frame/sec by confocal microscopy on Stage Top Incubator. The tip of the needle (Femtotips II, eppendorf) was broke off and its inner diameter adjusted to 3–4 μm was used for microinjection. The needle was inserted into enteroid by using Coarse and fine Three-axis Oil Hydraulic Micromanipulator and One-axis Oil Hydraulic Micromanipulator (MN-4, MMO-202ND, MMO-220A, NARISHIGE), and fluorescein, PBS, 1–10 mg/mL LPS from *S*.Typhimurium and 1 × 10^10^ CFU/mL *S*. Typhimurium *ΔphoP* were introduced to enteroid lumen by using Pneumatic Microinjector (IM-11–2, NARISHIGE). Fluorescence intensity of enteroid lumen and medium was measured by NIS-Elements AR. After microinjection, percent area granule secretion was calculated for ten Paneth cells in five enteroids for each sample. The delay time of Paneth cell secretion by microinjection was measured as the elapsed time from the start of introducing reagents into the enteroid lumen until the first granule was secreted.

### Volume measurement of enteroid lumen and microinjection

Enteroids at day 3 of culture were treated with 1 μM Rh123 (Sigma-Aldrich) for 180 min at 37 °C and 1 μm Z-stack images were acquired. The luminal volume of enteroid was measured as accumulated Rh123 in enteroid lumen using by 3D measurement tool in NIS-elements AR. For measurement of injection volume, fluorescein and mineral oil (Sigma-Aldrich) were loaded into the needle in the order and the diameters of the interface between fluorescein and mineral oil before and after microinjection and moving distance by microinjection were measured.

### Statistical analysis

All statistical computations were performed using GraphPad Prism (GraphPad Software). Data comparing two groups were analyzed by two-tailed unpaired Student’s t-test. Data comparing several treatments were analyzed by one-way analysis of variance (ANOVA) followed by Tukey’s post hoc test for multiple comparisons. Correlation analysis was performed using Pearson correlation coefficient. Differences between groups were considered significant if P-values were < 0.05.

## Supplementary information

Supplementary Information

Supplementary Video S1

Supplementary Video S2

Supplementary Video S3

Supplementary Video S4

Supplementary Video S5

Supplementary Video S6

Supplementary Video S7

## Data Availability

The datasets generated during and/or analysed during the current study are available from the corresponding author on reasonable request.
