# Supplementary material for: Paneth cell granule dynamics on secretory responses to bacterial stimuli in enteroids
Source: Sci Rep. 2019 Feb 25;9:2710. doi: 10.1038/s41598-019-39610-7 (PMC6389922; doi:10.1038/s41598-019-39610-7)
Supplement: Supplementary file 1 — Supplementary Information [file 41598_2019_39610_MOESM1_ESM.pdf]

Supplementary Information

Paneth cell granule dynamics on secretory responses to bacterial stimuli in enteroids

Yuki Yokoi [a], Kiminori Nakamura [a,b], Tsukasa Yoneda [a], Mani Kikuchi [b], Rina Sugimoto [a], Yu Shimizu [a], and Tokiyoshi Ayabe [a,b,*]

[a] Innate Immunity Laboratory, Graduate School of Life Science and [b] Department of Cell Biological Science, Faculty of Advanced Life Science, Hokkaido University, Kita-21, Nishi-11, Kita-ku, Sapporo, Hokkaido 001-0021, Japan

*Corresponding author

Tokiyoshi Ayabe

Supplementary Table S1. Dilution ratio of introduced substances into enteroid lumen by microinjection

Volume of enteroid lumen (pL)	Injection volume (pL)	Dilution ratio
54.4±3.0	2.5±0.1	21.5±1.2

Volume of enteroid lumen: Mean volume of enteroid lumen calculated from Z-stack images of enteroid at day3 of culture stained the lumen by 1 μM Rh123. Injection volume: Mean volume of fluorescein discharged from the needle calculated from inner diameter and moving distance of liquid interface of mineral oil and fluorescein before and after microinjection. Dilution ratio of introduced substances into enteroid lumen by microinjection were calculated from volume of enteroid lumen and injection volume. The values were depicted as mean ± standard error of the mean in fifteen replicate experiments.

Supplementary Video S1. Paneth cell granule secretion induced by CCh

Time-lapse images of Paneth cells taken by confocal microscopy at 15 frame/sec after adding 10 μM CCh to the culture medium. Real acquisition time are represented by h:mm:ss (hour:minute:second). Scale bar: 10 μm.

Supplementary Video S2. Enteroid lumen microinjection of fluorescein

Time-lapse images of introducing fluorescein into enteroid lumen by microinjection. The video was taken at 15 frame/sec. Real acquisition time are represented by h:mm:ss (hour:minute:second). Scale bar: 50 μm.

Supplementary Video S3. Enteroid lumen stained by Rh123

Z-stack images of enteroid lumen stained by 1 μM Rh123 (Pink: lumen). 3D structure image (213.17 μm × 213.17 μm. Depth, 74.00 μm) was generated by NIS-Elements.

Supplementary Video S4. Paneth cell granule secretion in response to LPS from *S.* Typhimurium

Time-lapse images of introducing 10 mg/mL LPS from *S.* Typhimurium into enteroid lumen by microinjection taken at 15 frame/sec. Real acquisition time are represented by h:mm:ss (hour:minute:second). Scale bar: 10 μm.

Supplementary Video S5. Enteroid lumen microinjection of PBS

Time-lapse images of introducing PBS into enteroid lumen by microinjection taken at 15 frame/sec. Real acquisition time are represented by h:mm:ss (hour:minute:second). Scale bar: 10 μm.

Supplementary Video S6. Paneth cell granule secretion in response to *S.* Typhimurium *ΔphoP*

Time-lapse images of introducing 1 × 10^{10} CFU/mL *S.* Typhimurium *Δphop* into enteroid lumen by microinjection taken at 15 frame/sec. Real acquisition time are represented by h:mm:ss (hour:minute:second). Scale bar: 10 μm.

Supplementary Video S7. Granule replenishment of Paneth cells

Time-lapse images of Paneth cell (white arrow head) regenerating their granules after granule secretion induced by 1 μM CCh for 10 min taken at 4 frame/hr. After acquisition of Z-stack time-lapse images, the movie was rearranged by choosing the images in focus on granules at each time. Time 0 denotes the time frame immediately after washing out CCh. Real acquisition time are represented by h:mm:ss (hour:minute:second). Scale bar: 20 μm.
